# Comparative proteome analysis in an *Escherichia coli* CyDisCo strain identifies stress responses related to protein production, oxidative stress and accumulation of misfolded protein

**DOI:** 10.1186/s12934-019-1071-7

**Published:** 2019-01-29

**Authors:** Isabel Guerrero Montero, Katarzyna Magdalena Dolata, Rabea Schlüter, Gilles Malherbe, Susanne Sievers, Daniela Zühlke, Thomas Sura, Emma Dave, Katharina Riedel, Colin Robinson

**Affiliations:** 1 School of Biosciences, University of Kent, Canterbury, CT2 7NJ UK; 2 Institute of Microbiology, University of Greifswald, Felix-Hausdorff-Straße 8, 17487 Greifswald, Germany; 3 Imaging Center of the Department of Biology, University of Greifswald, Friedrich-Ludwig-Jahn-Str. 15, 17487 Greifswald, Germany; 4 UCB Celltech, 216 Bath Road, Slough, SL1 3WE UK

**Keywords:** *Escherichia coli* CyDisCo, Twin-arginine transport, Recombinant protein, Disulfide bond formation, Misfolding, Proteome, Stress response

## Abstract

**Background:**

The Twin-arginine translocation (Tat) pathway of *Escherichia coli* has great potential for the export of biopharmaceuticals to the periplasm due to its ability to transport folded proteins, and its proofreading mechanism that allows correctly folded proteins to translocate. Coupling the Tat-dependent protein secretion with the formation of disulfide bonds in the cytoplasm of *E. coli* CyDisCo provides a powerful platform for the production of industrially challenging proteins. In this study, we investigated the effects on the *E. coli* cells of exporting a folded substrate (scFv) to the periplasm using a Tat signal peptide, and the effects of expressing an export-incompetent misfolded variant.

**Results:**

Cell growth is decreased when either the correctly folded or misfolded scFv is expressed with a Tat signal peptide. However, only the production of misfolded scFv leads to cell aggregation and formation of inclusion bodies. The comprehensive proteomic analysis revealed that both conditions, recombinant protein overexpression and misfolded protein accumulation, lead to downregulation of membrane transporters responsible for protein folding and insertion into the membrane while upregulating the production of chaperones and proteases involved in removing aggregates. These conditions also differentially affect the production of transcription factors and proteins involved in DNA replication. The most distinct stress response observed was the cell aggregation caused by elevated levels of antigen 43. Finally, Tat-dependent secretion causes an increase in *tatA* expression only after induction of protein expression, while the subsequent post-induction analysis revealed lower *tatA* and *tatB* expression levels, which correlate with lowered TatA and TatB protein abundance.

**Conclusions:**

The study identified characteristic changes occurring as a result of the production of both a folded and a misfolded protein, but also highlights an exclusive unfolded stress response. Countering and compensating for these changes may result in higher yields of pharmaceutically relevant proteins exported to the periplasm.

**Electronic supplementary material:**

The online version of this article (10.1186/s12934-019-1071-7) contains supplementary material, which is available to authorized users.

## Background

*Escherichia coli* is a popular expression platform for the production of recombinant proteins of therapeutic interest due to the ease and relative low cost with which it can be rapidly grown and modified in controlled laboratory and industrial settings. Among its advantages over other cell factories we highlight the simplification of downstream processing if the protein of interest is exported to the periplasmic space. Since *E. coli* does not naturally export many proteins into the periplasm the recovery of the protein of interest will occur with minimal contamination of unwanted host cytosolic proteins, DNA or endotoxins [1].

The export of the recombinant protein to the periplasmic space most typically occurs via the Sec transport pathway ‘naturally’ and in most industrial applications. In this pathway, an unfolded substrate with a cleavable N-terminal signal peptide is recognized and transported through the membrane-bound Sec translocase to the periplasm where it is then folded to a functional state. This is also the primary means of producing disulfide-bonded proteins due to the oxidizing environment of the periplasm [2, 3]. One of the major disadvantages of this system is the inability of the Sec pathway to successfully export highly complex proteins that rapidly fold in the cytoplasm before they reach the translocase or that have post-translational modifications such as cofactor insertions, which is the case of many biotechnologically relevant proteins. To circumvent this problem another transport pathway that transports fully folded proteins exists: the Tat system. This system, named for the Twin arginine motif highly conserved in the signal peptides that localize the proteins to the transporter [4], has been shown not only to transport folded proteins from the cytoplasm to the periplasm [5, 6], but also to have a useful although poorly understood proofreading mechanism that blocks the export of proteins unless they are tightly folded and have the appropriate co-factors in place. By exploiting this system, heterologous proteins harvested from the periplasm will potentially contain a very low degree of protein heterogeneity, making it easier and more cost effective for downstream processing after harvest. Moreover, this export system is naturally capable of transporting proteins up to 150 kDa [7] which potentially allows the transport of relatively complex proteins [8]. The Tat system has also an important role in maintaining cellular homeostasis in *E. coli* [9].

Nevertheless, many proteins of biopharmaceutical interest are too complex to fully fold in the cytoplasm because disulfide bonds cannot properly form in the non-oxidizing cytoplasm of *E. coli*. There are several routes available to avoid this problem, either by expressing proteins that fold tightly without the need of disulfide bonds [10] or by using strains with an oxidizing cytoplasm [11, 12]. One of these strains is the CyDisCo (Cytoplasmic Disulfide bond formation in *E. coli*) strain which expresses a yeast mitochondrial thiol oxidase, Erv1p and the human protein disulfide isomerase, PDI both lacking secretion signals [13]. The expression of these two proteins allows the formation of disulfide bonds in the cytoplasm, which leads to the correct folding of the protein and thus to its export to the periplasm via the Tat pathway [14].

In this study we sought to understand the physiological changes that result from the expression of a recombinant protein in both its correctly folded state and in a deliberately misfolded state using an *E. coli* CyDisCo platform. The expression of heterologous proteins in *E. coli* often results in protein misfolding and aggregation because the cell is not able to sustain non-physiological rates of expression. For its industrial application, the production process must be optimized to achieve high protein yields. Thus, under protein production process conditions it is important to identify stress parameters to circumvent and improve the process efficiency. For this reason, we were interested in characterizing the changes that occur during the expression of a misfolded Tat substrate, in order to understand whether additional responses occur. The substrate used was a single chain variable fragment (scFv) construct that is efficiently exported by Tat while the ‘misfolded’ variant contains a 26-residue C-terminal extension that blocks export by Tat [14]. Both proteins were synthesised with an N-terminal TorA signal peptide that directs export by Tat. For our study, we used a comprehensive proteomic approach to monitor the changes in cell physiology and proteome caused by recombinant protein secretion, misfolding and aggregation.

## Methods

### Bacterial strains and plasmids

The *E. coli* strain utilized for this study was W3110. All plasmids (Table 1) are derived from pET23 and have a Ptac promoter before the signal peptide or CyDisCo components and a 6xHis-tag after the scFv and mutated scFv. pIGM01 was constructed by PCR amplification using pHAK13 as a template and primers Empty_CyD_F (5′-GAAGGAGATATTATGAAAGCCATCG-3′) and Empty_CyD_R (5′-CACCTAAACGGACTGGCTGTTT-3′) as forward and reverse primers. PCR solutions were made using 1 μL template DNA (100 ng/μL), 1 μL dNTPs (200 μM final), 10 μL 5× GC buffer, 1.25 μL of each primer (0.5 μM final), 0.5 μL Phusion High-fidelity DNA polymerase (2 U/μL stock, New England Biolabs, UK) and made up to a final volume of 50 μL with milliQ H_2_O. PCR was then carried out in Biometra T3 Thermocycler (Biometra Anachem, UK) as per Phusion polymerase instructions with an annealing temperature of primer T_m_ minus 5 °C and annealing time extended to 10 min. PCR product was then treated with 1 μL DpnI restriction enzyme (New England Biolabs, UK) for 1 h at 37 °C to digest template DNA and then heat inactivated for 20 min at 80 °C. 2 μL of the product were mixed with 1 μL of ligase buffer, 1 μL of T4 DNA ligase (Roche) and made up to a final volume of 10 μL with milliQ H_2_O before incubation overnight at 4 °C. The next day 5 μL ligation product was used to transform 100 μL *E. coli* W3110 competent cells. Final plasmid sequence was confirmed by GATC-Biotech before use in this study.

**Table 1:** Plasmids used in this work

Plasmid	Function	References/source
pHAK13	TorA-scFvM wild type with CyDisCo	[14]
pAJ22	TorA-scFvM-C-term26 (additional—SNAIIIIITNKDPNSSSVDKLAAALE′ 3′ to C-terminus) with CyDisCo	[14]
pIGM01	CyDisCo	This study

### Cell culturing, sampling and fractionation

5 mL Luria–Bertani (LB) medium (10 g/L sodium chloride, 10 g/L tryptone, 5 g/L yeast extract) pre-cultures were inoculated from glycerol stocks and grown aerobically overnight at 30 °C, 200 rpm with 1:1000 antibiotic (5 μL, 1 M ampicillin). The next day cultures were diluted to OD_600_ of 0.05 in 50 mL fresh LB and antibiotic and set in triplicate. Cultures were then grown at 30 °C, 200 rpm in 250 mL Erlenmeyer flask and samples were taken every 30 min for an OD_600_ reading. At OD_600_ of 0.4–0.6 plasmids were induced with 0.5 mM IPTG (25 μL, 1 M IPTG). After 3 h induction, cells equivalent to a density of OD_600_ of 10 (~ 8 mL) were taken and fractioned into cytoplasmic (C), insoluble/membrane (M), periplasmic (P) and inclusion bodies (IBs) samples. Periplasmic (P) fractions were collected using an EDTA/lysozyme/cold osmotic shock method previously described [15], with modifications [16]. Spheroplasts were further fractionated into inclusion bodies by centrifugation at 5,000 rpm for 15 min at 4 °C before separating the cytoplasmic (C) and insoluble/membrane (M) fractions as described earlier [16]. Fractions of three biological replicates from each strain were prepared.

The specific growth rate μ was calculated from three consecutive OD_600_ measurements and using the time points of 1.5 h as the initial point and 4.5 h as the final point of the exponential phase. The growth rate was calculated as previously described by McKay et al. [17]:

$$\mu = \frac{{\left( {ln\,x_{2} - ln\,x_{1} } \right)}}{\Delta t}$$

where *x*_*1*_ and *x*_*2*_ are the absorbance values measured at 600 nm at 1.5 h and 4.5 h, respectively.

Doubling time for the exponentially growing cultures was calculated as:

$$t_{d} = \frac{ln\,2}{\mu } .$$

### Protein expression analysis by Western blot

Protein was transferred to PVDF-membrane (GE Healthcare, Buckinghamshire, UK) by wet-western-blotting, with electrophoresis for 1 h at 80 V, 300 A. The PVDF membrane was then immersed in blocking solution (2.5% (w/v) skimmed milk powder in 50 mL 1× PBS-Tween20 (0.1%) and incubated at 4 °C overnight. The following day the membrane was washed 3× for 5 min with 1× PBS-Tween20 (0.1%) before incubation with primary antibody (3.5 μL anti-6-His (Life Technologies, CA, USA) in 20 mL 1× PBS-Tween20 (0.1%), 3% (w/v) BSA) for 1 h at room temperature. Membranes were then washed 3× for 5 min with 1× PBS-Tween20 (0.1%) before incubation with secondary antibody [4 μL anti-Mouse HRP conjugate (Promega, WI, USA) in 20 mL 1× PBS-Tween20 (0.1%)] for 1 h at room temperature. Finally, membranes were washed for 3× for 5 min with 1× PBS-Tween20 (0.1%). Immunoreactive bands were detected using an enhanced chemiluminescence (ECL) kit (BioRad, Herts, UK) following manufacturer’s instructions. Bands were visualised using BioRad Gel-doc chemiluminescence imager and associated software. Comparative densitometry of band-intensities was also carried out on a BioRad Gel-doc imager with an Image Lab™ Software 4.1.

### Transmission electron microscopy analysis

The cells were fixed (1% glutaraldehyde, 4% paraformaldehyde, 0.2% picric acid, 50 mM sodium azide in 20 mM HEPES buffer pH 7.4) for 5 min at 40 °C by using a microwave processor for laboratory use (H2500 Microwave Processor, Energy Beam Sciences Inc. East Granby, Connecticut, USA), and then for 30 min at room temperature. Finally, samples were stored overnight at 4 °C until further processing. Subsequent to embedding in low gelling agarose, cells were postfixed in 2% osmium tetroxide in washing buffer (20 mM cacodylate buffer pH 7, 10 mM calcium chloride) for 1 h at room temperature, followed by *en bloc* staining with 2% uranyl acetate in 0.05% sodium chloride for 30 min at room temperature and washing steps in between. After dehydration in graded series of ethanol (20%, 30%, 50%, 70%, 90% for 10 min each step, 96% two times for 10 min, 100% ethanol three times for 10 min) the material was embedded in AGAR 100 resin. Sections were cut on an ultramicrotome (Reichert Ultracut, Leica UK Ltd, Milton Keynes, UK), stained with 4% aqueous uranyl acetate for 3 min followed by lead citrate for 30 s and analyzed with a transmission electron microscope LEO 906 (Zeiss, Oberkochen, Germany). Afterwards, the micrographs were edited by using Adobe Photoshop CS6.

### Protein digestion and LC–MS analysis

The protein concentration was measured by the Bicinchoninic Acid (BCA) Protein Assay (Thermo Fisher Scientific). Cytoplasmic proteins (100 µg) were reduced with TCEP, alkylated with iodoacetamide and digested in-solution using trypsin as described by Muntel et al. [18]. Desalting of peptides prior to mass spectrometry analysis using Stage tips, C18 material (Thermo Fisher Scientific) was performed according to the protocol by Rappsilber et al. [19]. For the absolute quantification of proteins from cytoplasmic fraction, the peptide mix of a tryptic digest of yeast alcohol dehydrogenase (Waters, USA) was spiked to a final concentration of 50 fmol/μL. The nanoACQUITY™ UPLC™ system (Waters) was used to separate peptides and introduce the sample into the Synapt G2 (Waters) mass spectrometer. Parameters for liquid chromatography and IMS^E^ were used as described previously by Zühlke et al. [20].

Proteins from periplasmic and membrane fractions (30 μg) were separated via 1D SDS-PAGE and the protein staining was followed by Bradford method [21]. The entire gel lanes were cut into ten pieces each and proteins digested with trypsin (Promega, USA) overnight. Peptides were purified using ZipTip C18 tips (Millipore). The eluted peptides were subjected to LC–MS/MS analysis performed on a Proxeon nLC 1200 coupled online to an Orbitrap Elite (Thermo Fisher Scientific) mass spectrometer. Peptides were separated on in-house self-packed columns [id 100 μm, od 360 μm, length 200 mm; packed with 3.6 µm Aeris XB-C18 reversed-phase material (Phenomenex)] in an 80 min nonlinear gradient from 1% acetonitrile and 0.1% acetic acid to 95% acetonitrile, 0.1% acetic acid. A full MS scan (resolution of 60,000) was acquired using the automatic data-dependent mode of the instrument. After acquisition of the full MS spectra, up to 20 dependent scans (MS/MS) were performed according to precursor intensity by collision-induced dissociation fragmentation (CID) in the linear ion trap. The mass spectrometry proteomics data have been deposited to the ProteomeXchange Consortium (http://proteomecentral.proteomexchange.org) via the PRIDE partner repository with the dataset identifier PXD010078 (username: reviewer91495@ebi.ac.uk, password: gZ9aEwDr).

### Protein identification and quantification

The MS^E^ spectra of cytoplasmic samples were processed using PLGS v 3.0 and searched against a randomized *E. coli* K12 W3110 UniProt/Swissprot database (Proteome ID: UP000000318, 4 257 entries, version December 2017) with added amino acid sequences of scFv, mutated scFv, yeast mitochondrial thiol oxidase, Erv1p and human protein disulfide isomerase, PDI, laboratory contaminants and yeast ADH1 sequence. Processing and search parameters were described earlier [20]. MS/MS spectra of periplasmic and membrane samples were searched against the above-mentioned database, excluding yeast ADH1, using MaxQuant software (version 1.5.8.3) [22]. Peptide search was performed with the Andromeda search algorithms [23]. For positive protein identification the following criteria had to be met: a minimal peptide length of six amino acids, up to two missed cleavages, carbamidomethylation of cysteine specified as a fixed modification, N-terminal protein acetylation and methionine oxidation were set as variable modifications. The false discovery rate (FDR) was estimated and protein identifications with FDR < 1% were considered acceptable. A protein had to be identified in at least two out of 3 biological replicates, and a minimum of two unique peptides per protein was required for relative quantification using the label free quantification (LFQ) algorithm provided by MaxQuant. The Student’s t test requiring a *P* value of < 0.05 was subsequently conducted to determine proteins with significant alterations in protein abundance. Changes in protein abundance of the *E. coli* CyDisCo strains expressing the protein of interest vs. wild type (WT) were presented with a log2 fold change (FC). If proteins were quantified in only one of the strains, and were identified in three biological replicates, they were added to the list of identified ON/OFF proteins.

### Functional analysis and data visualization

The protein biological functions were assigned based on their TIGRfam roles according to The SEED (http://pubseed.theseed.org/) [24] complemented with manual curation. If the protein was identified in more than one fraction, the subcellular localization was assigned based on the *E. coli* specific STEP database (STEPdb) [25]. Voronoi treemaps were created using the Paver software (DECODON GmbH) [26].

### Transcriptional analysis

The cells were harvested right before induction, 30 min and 3 h after induction, and then normalized using the absorbance measurement. The total RNAs were extracted using RNeasy Plus Mini Kit (Qiagen) with RNAprotect Bacteria Reagent (Qiagen) according to the manufacturer’s instructions. The reverse transcription was performed using SuperScript IV Reverse Transcriptase (Invitrogen). Finally, the real-time PCR was run in a QuantStudio 7 Flex (Applied Biosystems) using TaqMan Gene Expression Master Mix in a 20 µL reaction volume with the protocol: 10 min at 95 °C and 40 cycles of 15 s at 95 °C, 1 min at 60 °C. The gene *rrsH* encoding 16S ribosomal RNA was used as a reference and the ΔΔC_t_ was averaged over triplicate measurements.

### Phase contrast microscopy

Cells were grown in LB medium with ampicillin up to OD_600_ of 0.5 and after that induced with 0.5 mM IPTG. Next, cells were incubated for 3 h at 30 °C and 180 rpm. For microscopy 10 μL bacterial culture were pipetted onto a thin layer of 1.5% agar (Sigma-Aldrich). Samples were photographed with an AxioCam MRm (Zeiss) camera mounted on an Axio Imager 2 (Zeiss) fluorescence microscope through an EC Plan-Neofluar 100×/1.3 oil objective. Images were acquired with ZEN 2011 software and exported as TIFF file format.

### Aggregation assay

Cells were assessed for cell aggregation by monitoring of sedimentation. Overnight cultures of the strains were adjusted to the same OD_600_ and grown to an OD_600_ of 0.5. Subsequently, bacterial cultures were induced with 0.5 mM IPTG and grown for another 3 h. Next, 5 mL samples were placed in sterile 10 mL tubes and 1 mL samples were transferred to a cuvette and covered with Parafilm. Samples were always prepared in 3 replicates each and incubated for 24 h at 30 °C without shaking. Images were acquired using a digital camera. Percentage aggregation was measured as previously described [27].

## Results and discussion

### Physiological characterization of scFv-secreting CyDisCo strains

**Fig. 1:**
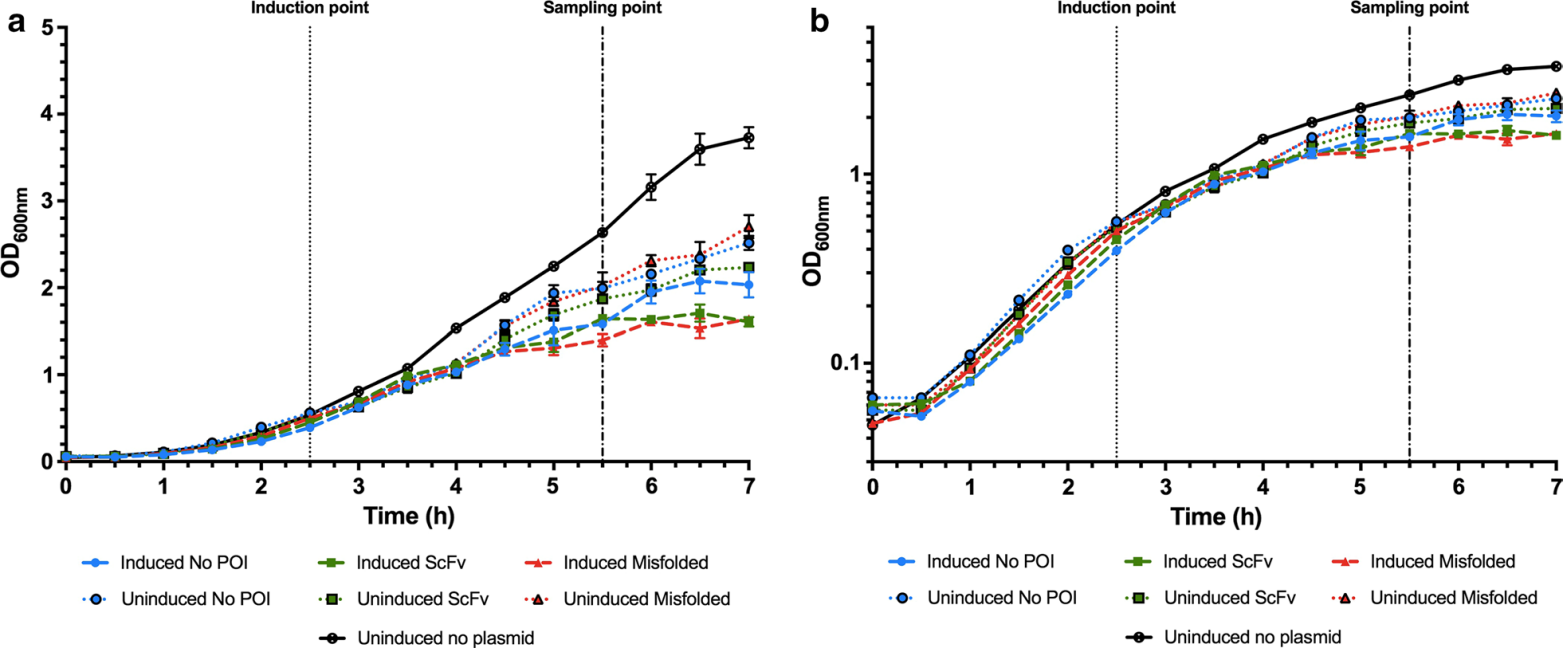
Growth comparison of *E. coli* CyDisCo induced and uninduced strains expressing folded, misfolded and no protein of interest (POI) as well as a control without plasmid. Linear (**a**) and semilogarithmic (**b**) display of *E. coli* growth. Cultivations were performed at 30 °C and 200 rpm for all conditions. Average growth and standard deviation of at least three independent experiments are shown. The dashed and dotted lines show the time of IPTG induction and sampling point (3 h post-induction), respectively

**Fig. 2:**
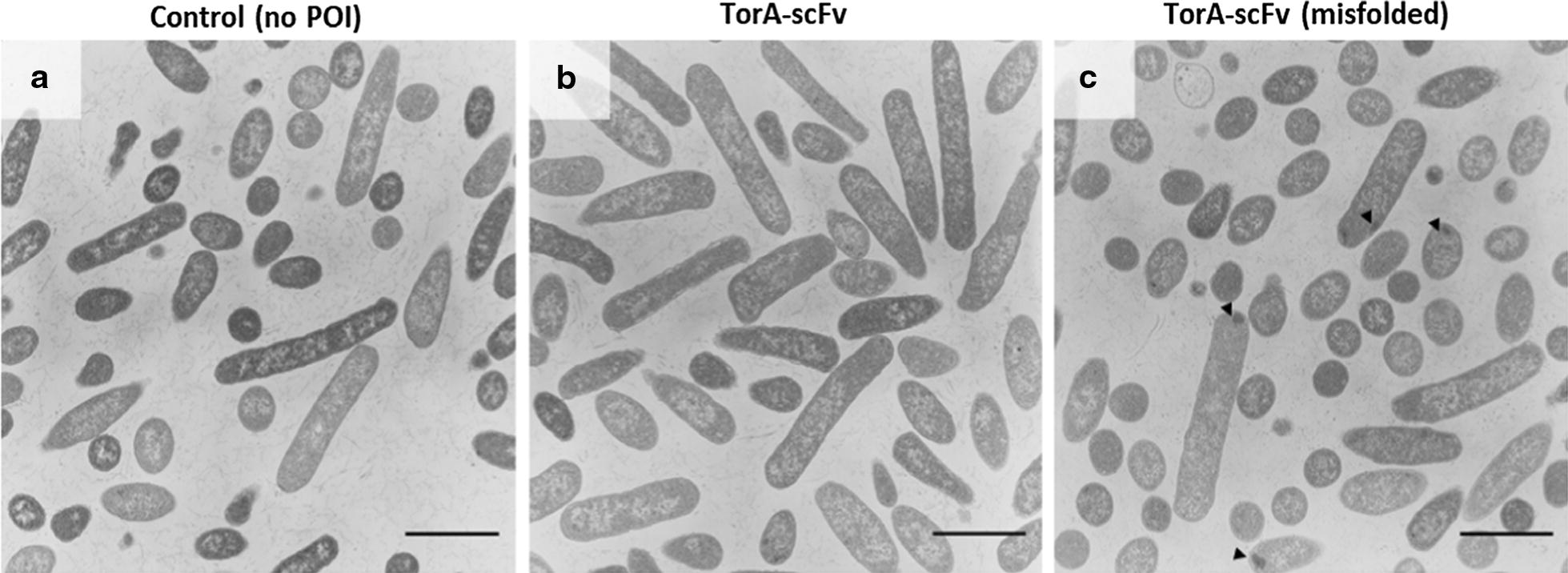
Transmission electron micrographs of *E. coli* CyDisCo: control (**a**), scFv (**b**) and misfolded scFv (**c**) expressing strains. The cells were grown aerobically in LB medium for 3 h post-induction with IPTG. The strain expressing misfolded TorA-scFv shows the formation of inclusion bodies (marked with arrowheads). Scale bar = 2 µm

A first indication of stress in scFv expressing cells was the metabolic burden. Metabolic burden occurs when the expression of a recombinant protein causes a decrease in generation time by diverting the resources of the cells towards protein production and plasmid maintenance [28]. Under the same growth conditions, we observed that the cells reach stationary plateau at different ODs when the plasmid is present and induced (Fig. 1). These different conditions tax the cells in different ways: when induction occurs, the cells reached the stationary plateau at a lower OD while expressing the protein of interest in both folded and misfolded state in comparison to those expressing only the CyDisCo components. In case of the cells producing the correctly folded scFv, this additional growth stress is most likely due to the recombinant protein synthesis and its export to the periplasm of the protein: although Tat transport does not consume ATP it uses the proton motive force to export the proteins to the periplasm [29], causing an energetic and resource deficit in comparison to the control. In case of the misfolded scFv, the resources are diverted to the synthesis of a recombinant protein in a large quantity, which unlike the correctly folded scFv, is not secreted to the periplasm but deposited in inclusion bodies (visible in Fig. 2c). When embedded in IBs, protein is devoid of any biological activity and primarily cannot be used again in the cell. Cells lacking a plasmid and hence not producing a heterologous protein, reached a maximum OD significantly higher than that when expressing recombinant protein. In summary, the highest metabolic burden is experienced by the cells producing both the CyDisCo components and the protein of interest (POI), however there is no clear difference between cells producing the correctly folded scFv and those producing the misfolded protein. Although the doubling times for all the strains were very similar (Table 2) we could observe that the lowest metabolic burden occurs when the cells do not have a plasmid followed by cells containing a non-induced plasmid since these reached much higher ODs.

**Table 2:** Comparison of the growth rate and doubling time of the exponential phase and maximum OD reached by the cultures after 7 h

Strains	Specific growth rate* (h^−1^)	Doubling time* (h)	Maximum OD (at 600 nm)
Induced no POI	0.75 ± 0.018	0.92 ± 0.022	2.08
Induced ScFv	0.74 ± 0.005	0.94 ± 0.006	1.71
Induced misfolded	0.69 ± 0.013	1.01 ± 0.019	1.64
Uninduced no POI	0.66 ± 0.022	1.05 ± 0.035	2.52
Uninduced ScFv	0.69 ± 0.019	1.01 ± 0.028	2.23
Uninduced misfolded	0.72 ± 0.016	0.96 ± 0.022	2.70
Empty	0.76 ± 0.018	0.91 ± 0.021	3.73

Cultivations were performed at 30 °C and 200 rpm for all conditions. The cells were grown aerobically in LB medium for 7 h and induced with IPTG after 2.5 h. The data for the calculation of the growth rate and generation time during the exponential phase was taken from time points at 1.5 h and 4.5 h

* Average from three replicates, ± standard deviation

**Fig. 3:**
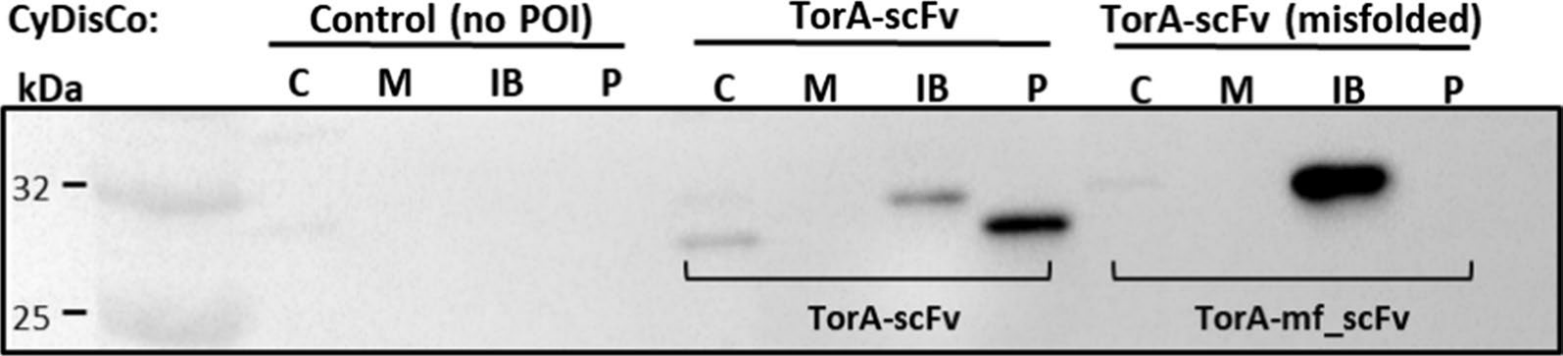
Accumulation of the scFv construct and its misfolded variant in CyDisCo cells. TorA-scFv and the misfolded TorA-scFv constructs were expressed in *E. coli* cells together with Erv1p and PDI, CyDisCo components. After expression, cells were fractionated into cytoplasmic (C), membrane (M), periplasmic (P) and inclusion body (IB) samples and extracted proteins separated via SDS-PAGE

For the proteomic study the time point of 3 h post induction was chosen since this coincided with the complete growth arrest for the cells producing the correctly folded scFv (Fig. 1) and, at the same time, provided satisfactory yields of recombinant protein (Fig. 3).

### Total expression of Tat-targeted scFv and misfolded scFv

The misfolded scFv (mf_scFv), consisting of an additional 26-residue unstructured tail at the C-terminus of the folded scFv, is not transported to the periplasm since the Tat system’s proofreading mechanism detects it as unfolded [14]. Figure 2c shows that IBs are formed in the cells expressing the unfolded protein, while Fig. 3 shows that the unfolded scFv is also unprocessed: the signal peptide has not been cleaved and hence the protein runs higher on the SDS gel. A similar phenomenon occurs with the correctly folded scFv since a fraction of the uncleaved protein is also found in inclusion bodies, whereas the bulk of the protein is correctly exported to the periplasm and a small proportion remains in the cytoplasm. In both cases we can observe that there is less accumulation in inclusion bodies for the folded scFv and a densitometry analysis shows that the protein present in inclusion bodies in the unfolded scFv is 6.86× more abundant than that of the folded. This accumulation in IBs in the cytoplasm may be due to the low abundance of Tat machinery available to transport the protein across the inner membrane, since over-expression of the Tat components leads to more efficient export [30]. Finally, it has been previously shown and discussed that the presence of the mature-sized scFv in the cytoplasm may be due to early cleavage taking place by cytoplasmic peptidases [10].

### Impact of elevated scFv secretion and misfolded scFv accumulation on the *E. coli* proteome

In the previous section, we showed that the expression of TorA-scFv in CyDisCo cells results in a high secretion of the protein to the periplasm and minor accumulation in IBs, whereas the misfolded version aggregates in the IB fraction. Both processes are likely to cause some similar proteome stress responses associated with recombinant protein production, but we were most interested to determine whether diverse responses occurred due to the intensified secretion of scFv and its accumulation in the periplasm, or an aggregation of oxidized misfolded scFv in the cytoplasm.

**Fig. 4:**
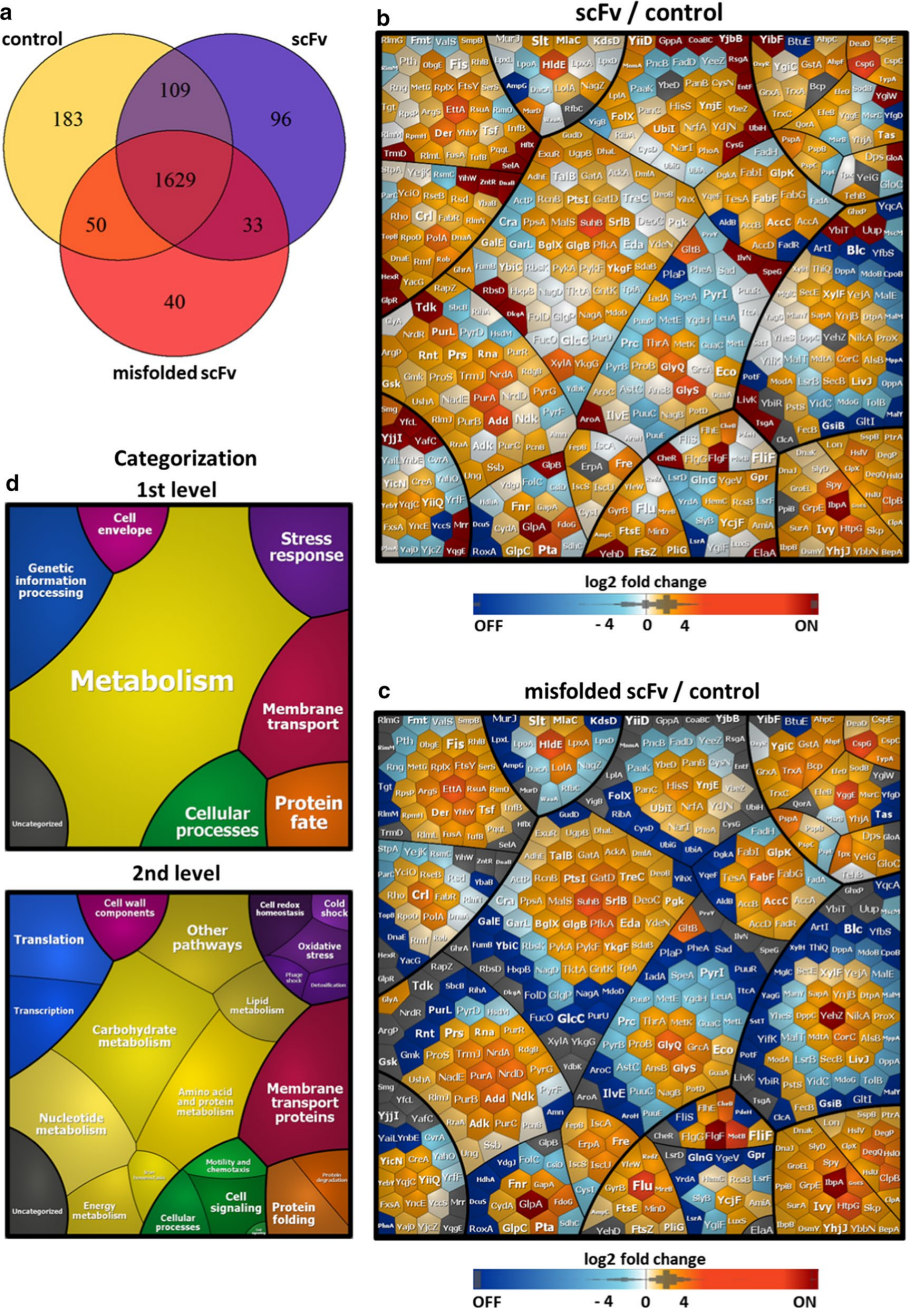
Analysis of *E. coli* CyDisCo proteome changes in the strains expressing scFv and misfolded scFv vs. an empty plasmid control. Venn diagram showing the number of unique and shared proteins identified in *E. coli* CyDisCo strains (**a**). Proteome changes are shown as the change in protein abundance (Δlog2 ratio) of strains expressing: TorA-scFv vs. control (**b**) and TorA-mf_scFv vs. control (**c**). Changes in protein abundance were color coded: orange indicates increase in the scFv and scFv (misfolded) proteome, gray indicates unidentified, and blue indicates decrease in the scFv and scFv (misfolded) proteome. Additionally, proteins which were identified in scFv expressing strains but not in the control strain were colored red (ON proteins) and proteins identified only in control strain but not in scFv expressing strains were colored navy blue (OFF proteins). The treemap legend shows the classification of the *E. coli* proteome according to TIGRfam annotations with manual adjustments (**d**)

To study the stress responses, we performed a subcellular fractionation and applied LC-IMS^E^ and LC–MS/MS approaches for a comprehensive proteome analysis. Quantification of cytoplasmic, periplasmic and membrane/insoluble proteins was performed for samples harvested 3 h post-induction (Additional file 1: Table S1). In our study we identified a total of 1629 proteins shared between three studied *E. coli* strains (Fig. 4a). Among these, 441 proteins of the strain secreting scFv and 393 proteins of the strain expressing mf_scFv exhibited an at least twofold change in expression when compared to the control (Additional file 2: Table S2). Changes in the amount of proteins are visualized by Voronoi treemaps (Fig. 4b–d). The proteins are clustered by their functional category (TIGRfam complemented with manual curation) so that functionally related gene products are localized in one cluster. We distinguished several patterns of changes in the abundance of functionally related proteins and described them in the following section, providing log2 FC ratios for statistically significant results (p < 0.05). The first conclusion is that the expression of misfolded protein results in downregulation of many proteins involved in general metabolism and membrane transport. In addition, strains expressing either misfolded or correctly folded scFv, showed a high number of significant changes mostly in proteins associated with protein folding and degradation, oxidative stress, membrane transport and integrity.

### Folded vs. misfolded scFv stress response affecting transcription factors and DNA replication

The adaptation to changes in growth conditions is often regulated by alternative sigma factors and anti-sigma factors [31]. Sigma factors are multi-domain subunits of the bacterial RNA polymerase and essential for the initiation of transcription. They are involved in the recognition of promoters and participate in the initial steps of RNA synthesis [32]. The proteomic analysis revealed that there was an increase in the levels of different sigma factors in the cells producing misfolded and folded scFv. This observation confirms that these cells are compensating for the additional production of the protein of interest by increasing the capacity of the cells to handle the metabolic burden. However, the results for the upregulation of transcription factors identified in our study failed to reach the desired level of statistical significance, thus they will be not discussed in detail.

Additionally, we observed an upregulation of many proteins involved in DNA replication occurring only in the cells producing the correctly folded TorA-scFv. For example, a single-stranded DNA-binding protein Ssb (1.46 log2 FC scFv vs. control) or even a protein as essential in DNA replication as PolA (DNA polymerase I) (0.85 log2 FC scFv vs. control in the cytoplasmic fraction) were found to be upregulated in scFv producing strain whereas the cells expressing mf_scFv showed no change. The upregulation of proteins involved in DNA replication only in cells producing the folded protein suggests that, even though according to the growth curve all three conditions seem to either have reached (scFv and mf_scFv) or be entering (control) the stationary phase, the strain producing folded scFv is maintaining expression of proteins involved in DNA replication, repair and recombination [33].

Finally, we would like to highlight the upregulation of RlmJ (previously known as YhiR) in both strains producing folded and misfolded protein (1.40 and 0.76 log2 FC, respectively). RlmJ is involved in the catabolism of external DNA [34] and has been identified to repress cell-to-cell plasmid transfer [35]. Notably, the abundance of RlmJ is lower in the cells producing mf_scFv, meaning that even though the misfolded protein stress seems to be partially responsible for the observed upregulation, this effect is greater with the production and transport of a folded protein to the periplasm.

### Protein folding and degradation processes

To assure the proteome stability and functionality during high-rate protein expression, *E. coli* activates the proteome quality control system termed the proteostasis network [36, 37]. This system consists of several complex protein machines that under stress conditions maintain proteome homeostasis by a proper regulation of protein folding and degradation processes. Here, we identified main chaperones and proteases upregulated upon scFv and mf_scFv expression in CyDisCo and describe their functional implication in proteome stability.

DnaK–DnaJ–GrpE, and GroEL–GroES are the best characterized molecular chaperones in the cytoplasm of *E. coli*. Both systems can recognize unfolded proteins, refold or direct them to the cellular protein degradation machinery [38]. We observed the upregulation of chaperones from above mentioned systems in the cytoplasmic fraction of *E. coli* expressing the recombinant protein. However, the highest abundance of these proteins was observed in the membrane fraction containing IBs, and the upregulation of GroES–GroEL and DnaK was notably increased in the strain expressing mf_scFv (GroES, 2.33; GroEL, 1.43; DnaK, 1.10 log2 FC mf_scFv vs. control). This observation correlates with our previous results and confirms the aggregation of moderate amounts of scFv in the cytoplasm and the high-level accumulation of mf_scFv in IBs. Moreover, the abundance of ClpB, a chaperone cooperating with DnaK to reactivate strongly aggregated proteins [39], was significantly increased in cells producing scFv and mf_scFv (2.39 and 2.84 log2 FC, respectively). The inclusion body formation in the CyDisCo scFv and mf_scFv strains was also confirmed by the presence of IbpA, a heat shock protein with a strong ability to associate to protein aggregates, that was not identified in the empty plasmid control strain, in which we did not find any traces of IBs. Additionally, several other chaperones such as HtpG involved in *de*-*novo* protein folding in the cytoplasm [40]; Spy (2.37 and 2.25 log2 FC) inhibiting protein aggregation and promoting refolding in the periplasm [41]; and Skp (1.44; 1.48), SurA (1.36; 1.24), BepA (1.46; 1.14) accelerating folding and improving insertion of outer membrane proteins [42, 43], were found to be highly abundant in the scFv and mf_scFv expressing strains, respectively. The accumulation of protein aggregates in the cytoplasm of *E. coli* CyDisCo is followed by activation of protein degradation machineries. We observed the upregulation of the ATPase components ClpA and ClpX of the Clp protease, Lon and HslV/U, together with periplasmic proteases DegP/Q. All these proteins are responsible for recognition, resolubilization and degradation of unfolded proteins. The highest difference between the abundance of scFv and mf_scFv expressing strains and the control was identified for periplasmic DegP and DegQ (1.53, 2.56; 1.85, 3.10 log2 FC for scFv and mf_scFv, respectively) proteases. This suggests that in both conditions, scFv secretion to the periplasm and mf_scFv aggregation in the cytoplasm, the cell envelope is exposed to unfavourable and harmful changes.

The high upregulation of periplasmic proteases in the strain expressing misfolded scFv is particularly interesting. This could indicate that some of the protein is in fact successfully exported to the periplasm, rather than being blocked by the Tat proofreading machinery, and subsequently degraded. However, a previous study showed no evidence whatsoever for transport of this misfolded substrate to the periplasm and in our view, it is more likely that the cell either senses the production of precursor protein and upregulates expression, or the proteases are upregulated as part of a general stress response.

### Oxidative stress and redox regulation in the cytoplasm

The principle behind disulfide bond formation in the CyDisCo strain, as described before, is the co-expression of Erv1p and PDI in the cytoplasm. This system allows the oxidation of a dithiol to a disulfide in the cytoplasm, and at the same time does not require the disruption of any naturally occurring reduction pathways. The expression of two exogenous oxidases changes the cytoplasm into an oxidized environment, where the disulfide bonds can form efficiently, which is proven by the effective secretion of disulfide bond-containing scFv to the periplasm through the Tat system (Fig. 3). However, since the oxidative state of the cytoplasm does not occur naturally, we expected that this will provoke an oxidative stress response and changes in the abundance of redox proteins.

Indeed, the proteomic analysis revealed the upregulation of several proteins related to oxidative stress response. Among these, major thiol-disulfide oxidoreductases: thioredoxin TrxA, glutaredoxin GrxA, together with thiol-specific peroxidase Bcp, were upregulated in strains expressing scFv (with statistically significant upregulation only in mf_scFv). The upregulation of oxidoreductases was significantly higher in the strain producing misfolded scFv, probably due to the increased protein accumulation in IBs (e.g. GrxA, 1.19; TrxA, 2.02, Bcp, 2.08 log2 FC mf_scFv vs. control). In the cytoplasm of *E. coli*, thioredoxin and glutathione/glutaredoxin systems are required for the reduction of proteins that become oxidized, and thus they maintain the redox homeostasis [11]. However, during severe oxidative stress both systems can be overwhelmed, or their protein function can shift from reductase to oxidase, leaving cytosolic proteins susceptible to oxidation. It has been shown that thioredoxins and glutaredoxins can act as oxidases when exported to the oxidizing periplasm [44, 45] and both, TrxA and TrxC, shift their function to oxidases in a *trxB* mutant [46]. A related point to consider is the fact that in the periplasm of *E. coli*, which is an oxidizing environment, other thioredoxin-related proteins such as DsbA, B, C, D, G catalyze the disulfide-bond formation and rearrangement. Thus, it appears that the function of thioredoxins and glutaredoxins is influenced by the redox potential of the cellular environment and in an oxidized cytoplasm of *E. coli* CyDisCo they will promote disulfide bond formation, rather than their reduction.

In conclusion, the production of disulfide bond-containing scFv in CyDisCo increases the abundance of cellular redox proteins in the cytoplasm. We hypothesize that the abundance of those proteins is regulated by the amount of oxidized protein aggregates in the cytoplasm, thus it is larger in the mf_scFv strain. Since the oxidizing potential of the cytoplasm in CyDisCo strains is not compromised, we assume that the redox processes are either not efficient, or in the oxidizing environment the oxidoreductases function is shifted to thiol oxidases.

### Membrane transporters

It was interesting to note that the components of three major secretion machineries such as the Sec pathway, Tat system and YidC showed similar changes in their abundance in CyDisCo strains expressing scFv and its misfolded variant. Essentially, we might expect to find Tat components more abundant in the strain secreting high levels of scFv to the periplasm. However, we observed moderately lower levels of TatA and TatB, as well as a higher level of TatE in CyDisCo strains expressing scFv and mf_scFv. We elaborate on this point in the next section.

The amounts of Sec system components showed an increased abundance in strains producing recombinant protein, with a log2 FC higher than 1 for SecB and SecE. The Sec protein translocation pathway is involved in transport of most of the membrane-bound and secreted proteins (reviewed in [47]). The upregulation of Sec machinery is likely to be an effect of elevated oxidative stress in the periplasm and changes in the cell envelope, to which the cell is responding by secretion of cytoprotective stress proteins. The Sec system is responsible for insertion of a large number of proteins into the *E. coli* membrane, however some of the membrane proteins require YidC activity for their insertion [48]. Interestingly, in our samples YidC was found in lowered amounts in the *E. coli* secreting scFv (− 2.29 log2 FC) and mf_scFv (− 2.57 log2 FC). Additionally, we have also observed a drastic decrease in the amount of ABC transporter proteins responsible for the utilization of di- and tripeptides. In *E. coli*, the oligopeptide transport systems play a main role in nutrition, but the transported peptides also serve as substrates for integral membrane assembly and take part in the functioning of quorum-sensing pathways [49]. In this study, particularly the substrate-binding proteins (SBPs) of the main *E. coli* peptide transporters were present in much-decreased amounts (DppA, − 5.74, − 5.60; OppA, − 3.68, − 2.97 and MppA, OFF, − 3.04 log2 FC in scFv vs. control and mf_scFv vs. control, respectively). These SBPs recognize the substrate and subsequently deliver it to the translocator, thus the decrease in the SBPs amount reduces the peptide transport rate. Moreover, some essential components of other transport systems such as maltose (MalE/M/T/Y), glutathione (GsiB), Tol-Pal (TolB, CpoB) and lipid (Blc), or osmoregulated- periplasmic glucan (OPG) biosynthesis pathway (MdoB) were downregulated. One feature that these downregulated proteins have in common is their role in membrane protein synthesis and membrane maintenance under stress conditions. This suggests that the scFv expression in the *E. coli* CyDisCo causes the membrane impairment through a decrease in number of membrane transporters. We hypothesize that this observed decrease in membrane transporters abundance can be an effect of YidC downregulation, since it has an important role in membrane protein folding and insertion into the membrane [50, 51]. Generally, it is plausible that downregulation of so many transporter proteins involved in metabolite uptake is a consequence of previously mentioned metabolic burden that occurs in cells expressing scFv and mf_scFv. The non-stressed cells (empty plasmid control) are still in exponential phase, showing higher metabolic and biosynthetic activity than cells expressing POI, already in stationary phase at the harvesting time.

### Decrease of tat genes transcription when expressing a Tat substrate

**Fig. 5:**
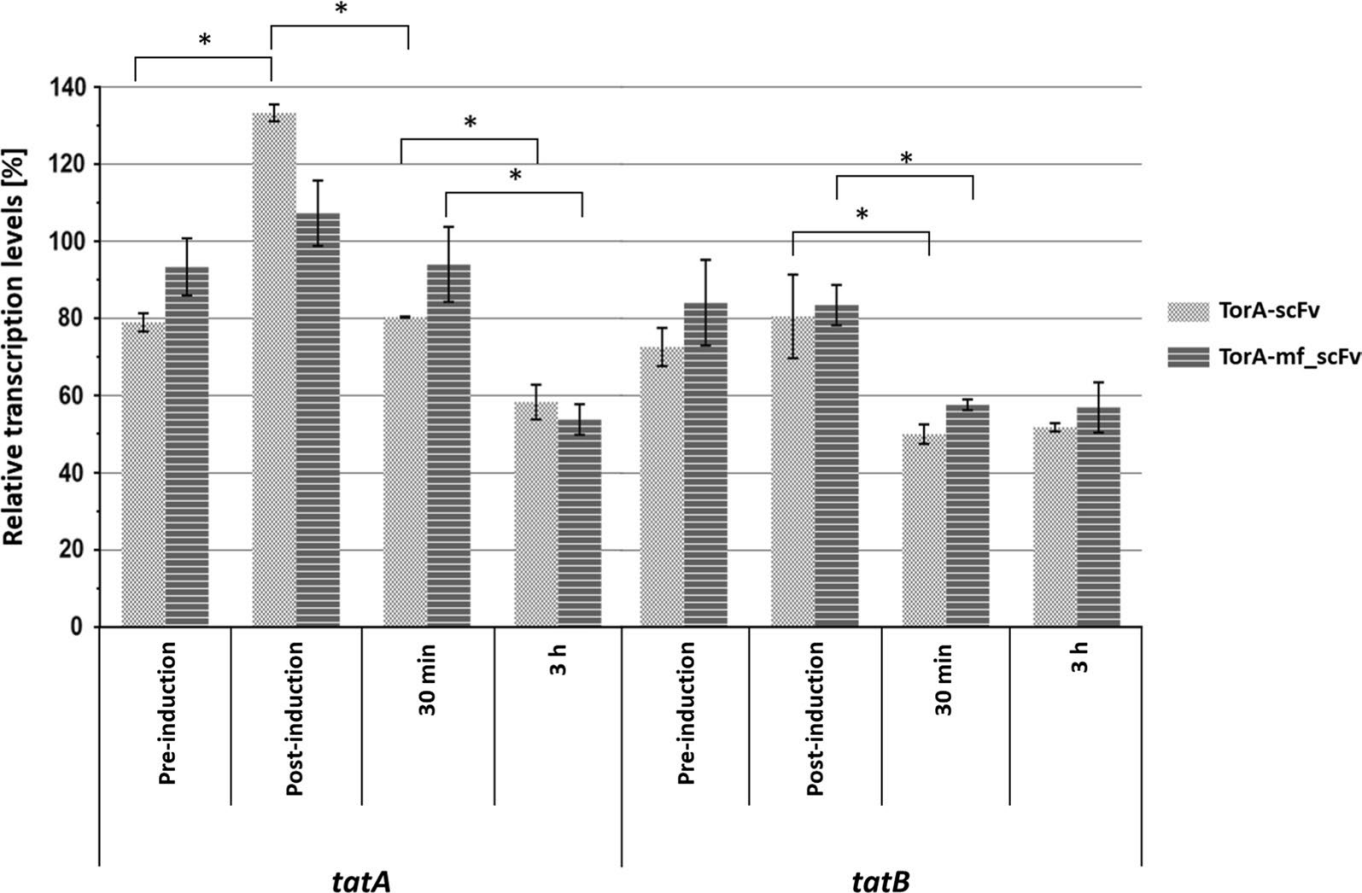
Transcription levels of *tatA* and *tatB* genes calculated from RT-qPCR measurements when expressing folded scFv (dark grey) or misfolded scFv (light grey) at four different time points after normalization to the control vector. The mean and standard deviations of three replicates are shown. *p < 0.02

The proteomic analysis showed a moderate increase in the abundance of TatE (0.37; 0.89 log2 FC for the scFv and mf_scFv vs. control, respectively) and a decrease of TatA and TatB proteins (however not statistically significant) in cells expressing scFv and misfolded scFv in comparison to the control. These observations raised the question of whether the expression of *tatA* and *tatB* genes is downregulated or the proteins have a shorter half-life upon overexpression of a Tat substrate. To address this question, an RT-qPCR experiment was conducted on the cells grown under the same conditions as for the proteomic study and reported relatively to the control (Fig. 5). A time course experiment was run including points just before and after induction, to assess the impact of IPTG on the cells. Only one significant change was observed due to induction; an increase of 69% was detected between pre- and post-induction samples for *tatA* expression with the folded scFv. Therefore, the induction has an immediate impact on expression level of *tatA* only when expressing a Tat-suitable substrate. Since the *tatABCD* genes are part of a single operon, this result means that the genes experience a differential transcription. Moreover, the higher transcription of *tatA* compared to *tatB* matches the previously established fact that *E. coli* contains more TatA than TatB proteins [52].

After induction, a significant decrease of transcription levels of *tatA* and *tatB* genes is observed when expressing TorA-scFv, whether correctly or incorrectly folded, by over 30%. This decrease matches the lower abundance of TatA and TatB proteins detected 3 h after induction. Therefore, less TatA and TatB proteins are expressed by the cell upon overexpression of a Tat substrate no matter whether it can fold correctly or not. These observations are surprising, given that the cells are expressing such high amounts of Tat substrate. The decrease in the amount of Tat operon being expressed seems to be partly compensated by the increase of TatE protein available in the cell. TatA and TatE share 53% homology and have overlapping functions. It has been shown that individual deletions of TatA and TatE are still functional, however the double deletion blocks export to the periplasm via the Tat pathway [53]. This preference for the use of either TatA or TatE is understudied and may be due to the additional stress conditions of the study or for a determined preference for the TorA signal peptide employed. Another explanation for this behaviour may be the fact that the Tat pathway exports few and complex proteins to the periplasm, many of them involved in cell division, biofilm formation and pathogenicity [7]. These proteins are in most cases not needed in high concentrations in the periplasm, and this negative feedback loop may be responsible for assuring that the export remains low to avoid toxicity in the periplasm.

### Misfolded scFv expression induces Ag43-mediated aggregation in *E. coli* CyDisCo cells

**Fig. 6:**
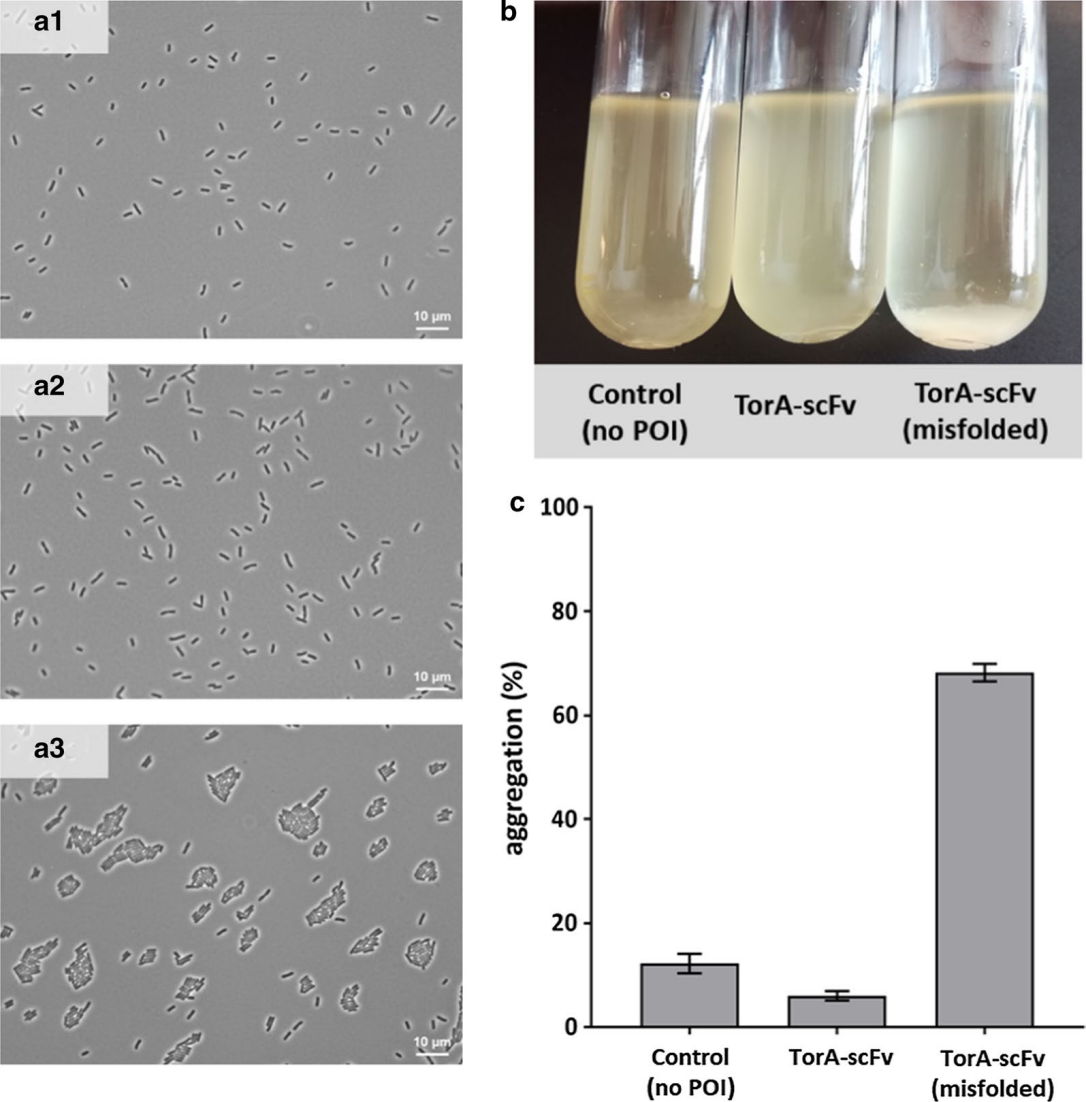
The *E. coli* CyDisCo strain expressing misfolded scFv shows Ag43-mediated cell aggregation. Phase-contrast microscopy images of CyDisCo: control (**a1**), scFv (**a2**) and misfolded scFv (**a3**) expressing strains. Samples were harvested 3 h post-induction with IPTG. **b** Sedimentation profile of *E. coli* CyDisCo strains. Cultures were grown for 3 h post-induction, transferred into tubes and incubated statically for 24 h. **c** Percentage of aggregation as quantified from the change in OD_600_ over 24 h from the experiment performed as in **b**

Our proteome studies revealed significantly higher (5.71 log2 FC) abundance of antigen 43 (Ag43) in the strain expressing a misfolded scFv. Ag43 is an outer membrane protein that confers bacterial cell-to-cell aggregation [54]. To our knowledge, the formation of cell aggregates connected with Ag43 expression has not yet been reported as a consequence of a cellular stress caused by the accumulation of misfolded protein in the cytoplasm. To verify if Ag43 upregulation promotes the cell aggregation in our strain, we first performed phase-contrast microscopy (Fig. 6a1–3). Additionally, since the cell aggregation leads to enhanced sedimentation, we applied a simple sedimentation assay (Fig. 6b) and calculated the aggregation rate in all CyDisCo strains (Fig. 6c). Indeed, a comparison of the aggregation and sedimentation rate of these strains confirmed that the *E. coli* expressing misfolded scFv shows enhanced sedimentation and aggregation. We propose that the increase in the abundance of Ag43 is related to the lowered abundance of the transcription factor OxyR, which is a suppressor of antigen 43 expression [55]. OxyR transcription factor is activated through the formation of a disulfide bond and is deactivated by enzymatic reduction with glutaredoxin 1, GrxA [56]. We observed a decrease in the abundance of OxyR in mf_scFv (− 0.67 log2 FC) and its upregulation in scFv expressing strain. These results could only be observed after the induction of the scFv expression, thus we assume that the accumulation of misfolded scFv leads to the Ag43-mediated cell clustering. We believe that the upregulation of Ag43 synthesis is related to the accumulation of oxidized misfolded scFv which perturbs the thiol-disulfide balance in the cytoplasm and causes a situation referred as ‘disulfide stress’. Interestingly, the downregulation of OxyR was only observed in the mf_scFv expressing strain, in which we observed higher upregulation of redox proteins. This suggests that the activity of OxyR is responsive to the thiol-disulfide redox status of the cell, however its abundance in the cell is regulated by another, yet not known, mechanism. Additionally, the absence of CpxR, an antagonistic regulator of adhesion in *E. coli* [57], could contribute to the enhanced cell aggregation under misfolded protein expression stress. The aggregation phenomenon was not accompanied by the increase in bacterial motility in the strain expressing misfolded scFv (Additional file 3: Figure S1).

## Conclusions

The *E. coli* CyDisCo strain enables folding of disulfide bond-containing scFv in the cytoplasm and its secretion in high amounts to the periplasm via the Tat system. The expression of an unfolded scFv led to protein aggregation and inclusion body formation. Decreased cell growth, low abundance of proteins involved in DNA replication and sigma 70 factor in *E. coli* expressing a misfolded scFv when compared to a folded scFv suggest that the unfolded protein stress accelerates cell transition to a stationary phase. We confirmed that the recombinant protein overexpression triggers the upregulation of proteins involved in removing the aggregates, such as chaperones and proteases. The high abundance of GroEL–GroES, DnaK and ClpB in the insoluble fraction of *E. coli* expressing misfolded protein suggests their association with protein aggregates. We also observed that the formation of disulfide bonds in the cytoplasm of *E. coli* CyDisCo initiates the oxidative stress response and increases the abundance of redox regulation system proteins. This upregulation of thioredoxins and glutaredoxins was higher in the strain accumulating the misfolded oxidized scFv in the cytoplasm. Nevertheless, the redox protein ability to reduce oxidized proteins seems to be compromised by the oxidative environment of the cytoplasm.

Interestingly, we observed an extreme downregulation of membrane transporters which could be related to a major decrease in the abundance of YidC transport system, responsible for protein folding and insertion into the membrane. Surprisingly, a high rate of Tat-dependent secretion caused an increase in *tatA* expression only just after induction of synthesis. The following post induction analysis revealed lower *tatA* and *tatB* expression levels, which correlate with lower TatA and TatB protein abundance. This could suggest that the presence of the Tat signal peptide actively regulates the amount of Tat components being produced in a feedback loop to prevent an unnecessarily high concentration of Tat substrates in the periplasm. Finally, the most distinct unfolded protein stress response we observed was the cell aggregation caused by the elevated levels of antigen 43. Interestingly, to the best of our knowledge, this phenomenon has not been reported yet as a consequence of misfolded protein expression, which makes it an interesting topic for further investigation.

## Additional files

**Additional file 1: Table S1.** Average protein abundances and fold changes for proteins from cytoplasmic (A), periplasmic (B) and insoluble/membrane (C) fractions.

**Additional file 2: Table S2.** Voronoi Treemap of log2 fold changes in protein abundance (*E. coli* CyDisCo vs. scFv and *E. coli* CyDisCo vs. mf_scFv).

**Additional file 3: Figure S1.** The swarming motilities of *E. coli* CyDisCo strains and *P. aeruginosa.* A swarming motility assay was performed using *E. coli* CyDisCo with an empty plasmid; expressing scFv; expressing misfolded scFv (mf_scFv) and *P. aeruginosa* PA01 as a positive control. Swarming plates (0.3% agar in LB medium) were spotted with 10 µL of overnight cultures and incubated for 24 h at 37 °C. After 24 h the *E. coli* CyDisCo strains did not show signs of cell swarming, while *P. aeruginosa* showed a positive swarming phenotype. Assays were performed in triplicate. Figure shows representative results from 24 h incubation.

## Abbreviations

Tat: Twin-arginine translocation
CyDisCo: Cytoplasmic Disulfide bond formation in *E. coli*
scFv: single-chain variable fragment
WT: wild type
FC: fold change
C: cytoplasm
M: membrane/insoluble
P: periplasm
IB: Inclusion bodies
POI: protein of interest
mf_scFv: misfolded scFv
SBP: substrate-binding proteins
