# Supplementary figures and images for: Comparative proteome analysis in an Escherichia coli CyDisCo strain identifies stress responses related to protein production, oxidative stress and accumulation of misfolded protein
Source: Microb Cell Fact. 2019 Jan 29;18:19. doi: 10.1186/s12934-019-1071-7 (PMC6350376; doi:10.1186/s12934-019-1071-7)

## Slide 1
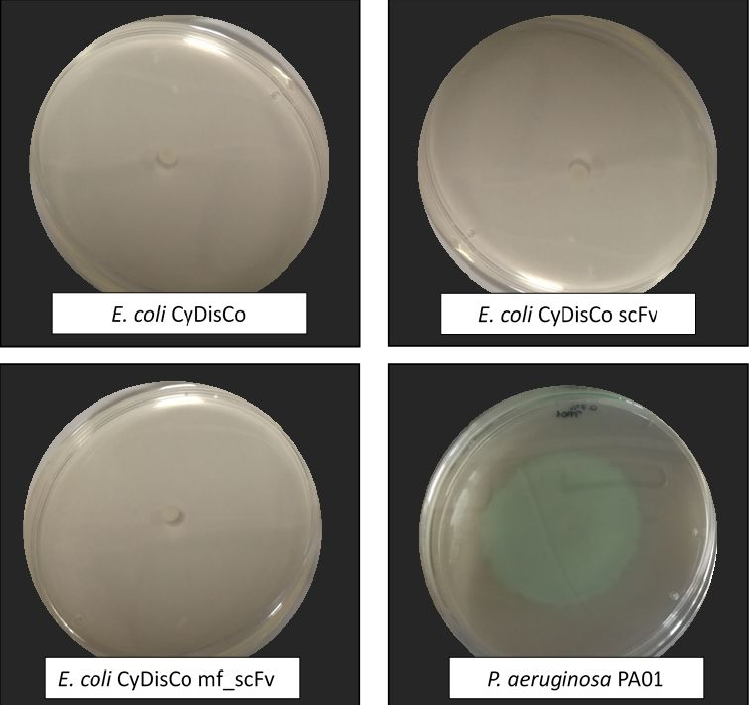

Supplement: Supplementary file 3 — Additional file 3: Figure S1. The swarming motilities of E. coli CyDisCo strains and P. aeruginosa. A swarming motility assay was performed using E. coli CyDisCo with an empty plasmid; expressing scFv; expressing misfolded scFv (mf_scFv) and P. aeruginosa PA01 as a positive control. Swarming plates (0.3% agar in LB medium) were spotted with 10 µL of overnight cultures and incubated for 24 h at 37 °C. After 24 h the E. coli CyDisCo strains did not show signs of cell swarming, while P. aeruginosa showed a positive swarming phenotype. Assays were performed in triplicate. Figure shows representative results from 24 h incubation. [file 12934_2019_1071_MOESM3_ESM.pptx]
